# Supplementary material for: Deletion of Macrophage-Specific Glycogen Synthase Kinase (GSK)-3α Promotes Atherosclerotic Regression in Ldlr−/− Mice
Source: Int J Mol Sci. 2022 Aug 18;23(16):9293. doi: 10.3390/ijms23169293 (PMC9409307; doi:10.3390/ijms23169293)
Supplement: Supplementary file 1 [file ijms-23-09293-s001.zip › ijms-1864061-supplementary.pdf]

Supplementary Material

1 Supplementary Data

Animals (in vivo studies)

Species	Vendor or Source	Background Strain	Sex
Mice	In House	C57	M
Mice	In House	C57	F

Genetically Modified Animals

Background Strain	Strain	Genotype
C57BL/6J	LiαKO	$Ldlr^{-/-}GSK3\alpha^{fl/fl}CsfiCre^{+/-}$
	LiβKO	$Ldlr^{-/-}GSK3\beta^{fl/fl}CsfiCre^{+/-}$
	LiαβKO	$Ldlr^{-/-}GSK3\alpha^{fl/fl}GSK3\beta^{fl/fl}CsfiCre^{+/-}$
	Lαfl/fl	$Ldlr^{-/-}GSK3\alpha^{fl/fl}$
	Lβfl/fl	$Ldlr^{-/-}GSK3\beta^{fl/fl}$
	Lαβfl/fl	$Ldlr^{-/-}GSK3\alpha^{fl/fl}GSK3\beta^{fl/fl}$

Antibodies

Target antigen	Vendor or Source	Working concentration
GSK-3α/β	Cell signaling	1:1000
GSK-3β	Cell signaling	1:1000
β-Actin	SIGMA	1:3000
CD107b(Mac3)	BD Transductions	1:50
CD68	Thermofisher Scientific	1:100
α-actin	Santa Cruz	1:100
NFKβ p65	Santa Cruz	1:50
IL-1β	Invitrogen	1:100
NLRP3	Invitrogen	1:100
CCR7	Abcam	1:100
ABCA1	NOVUS	1:100

2 Supplementary Figures and Tables

Supplementary Table S1. Metabolic Parameters of female mice

	Baseline	Neg Control	LiαKO		LiβKO		LiαβKO	
			Tam-	Tam+	Tam-	Tam+	Tam-	Tam+
Fasting Plasma Concentration, mM								
Cholesterol	10.65 ± 0.511	5.89 ± 0.274****	5.55 ± 0.190****	5.24 ± 0.228****	6.21 ± 0.467****	5.58 ± 0.249****	5.69 ± 0.227****	5.25 ± 0.212****
Triglyceride	2.71 ± 0.185	0.73 ± 0.052****	0.52 ± 0.033****	0.57 ± 0.046****	0.87 ± 0.089****	0.86 ± 0.060****	0.90 ± 0.064****	0.61 ± 0.037****
Blood Glucose,mM	11.28 ± 0.86	9.66 ± 0.43	9.53 ± 0.25	9.97 ± 0.51	11.05 ± 0.43	10.97 ± 0.33	9.35 ± 0.20	9.28 ± 0.38
Body weight, g	27.12 ± 1.24	29.73 ± 1.62	25.43 ± 1.06	25.11 ± 0.48	33.11 ± 1.74	29.00 ± 1.26	31.73 ± 1/06	27.51 ± 0.08
Liver weight, g	1.37 ± 0.11	1.20 ± 0.07	1.09 ± 0.04	1.02 ± 0.03	1.23 ± 0.06	1.10 ± 0.05	1.23 ± 0.04	1.10 ± 0.04
Adipose weight, g	0.49 ± 0.11	1.20 ± 0.21$^{\#\#}$	0.43 ± 0.08	0.19 ± 0.03$^{\#\#}$	1.40 ± 0.30	0.56 ± 0.12$^{\#\#}$	0.77 ± 0.16	0.30 ± 0.07$^{\#\#}$
Heart weight, g	0.14 ± 0.005	0.14 ± 0.004	0.16 ± 0.003	0.14 ± 0.003	0.14 ± 0.003	0.13 ± 0.003	0.15 ± 0.005	0.15 ± 0.005

Females, n=10-13 per group, *, in compare to baseline group #, in compare to non tamoxifen treated group

Supplementary Table S2. Metabolic Parameters of male mice

	Baseline	Neg Control	LiαKO		LiβKO		LiαβKO	
			Tam-	Tam+	Tam-	Tam+	Tam-	Tam+
Fasting Plasma Concentration, mM								
Cholesterol	8.26 ± 0.321	6.65 ± 0.387*	4.79 ± 0.205****	4.83 ± 0.221****	6.28 ± 0.130**	6.80 ± 0.139*	5.33 ± 0.411****	5.73 ± 0.286****
Triglyceride	2.35 ± 0.270	1.46 ± 0.185***	0.72 ± 0.047****	0.71 ± 0.055****	1.23 ± 0.066****	1.67 ± 0.089*	0.93 ± 0.106****	1.03 ± 0.093****
Blood Glucose,mM	11.18 ± 0.84	12.23 ± 0.70	9.79 ± 0.54	10.59 ± 0.36	11.51 ± 0.61	13.32 ± 0.57	10.67 ± 0.55	12.52 ± 0.37
Body weight, g	35.78 ± 1.48	43.57 ± 2.93	31.50 ± 0.89	31.26 ± 0.96	43.28 ± 2.50	43.18 ± 1.39	35.99 ± 1.02	35.60 ± 1.14
Liver weight, g	1.74 ± 0.12	1.96 ± 0.17	1.45 ± 0.07	1.44 ± 0.05	2.00 ± 0.25	2.05 ± 0.10	1.47 ± 0.08	1.61 ± 0.07
Adipose weight, g	1.19 ± 0.21	1.75 ± 0.30	0.37 ± 0.05	0.50 ± 0.05	1.92 ± 0.19	2.23 ± 0.14	0.80 ± 0.16	0.87 ± 0.12
Heart weight, g	0.19 ± 0.003	0.18 ± 0.003	0.19 ± 0.003	0.19 ± 0.006	0.17 ± 0.004	0.18 ± 0.002	0.19 ± 0.013	0.18 ± 0.003

Males, n=10-13 per group, *, in compare to baseline group

Supplementary Table S3. Mouse models

Mouse Line (C57BL/6J)		Tamoxifen	Deficiency ($Ldlr^{-/-}$)	Analyses
$Ldlr^{-/-}CsfiCre^{+/-}GSK3\alpha^{fl/fl}$	**LiαKO**	-	-	• GSK3α/β expression<br>• Atherosclerosis regression (aortic sinus)<br>• Characterization of plaques<br>• Lipid profiles
$Ldlr^{-/-}CsfiCre^{+/-}GSK3\beta^{fl/fl}$	**LiβKO**	-	-	
$Ldlr^{-/-}CsfiCre^{+/-}GSK3\alpha^{fl/fl}GSK3\beta^{fl/fl}$	**LiαβKO**	-	-	
$Ldlr^{-/-}CsfiCre^{+/-}GSK3\alpha^{fl/fl}$	**LiαKO**	+	macrophage GSK3α$^{-/-}$	
$Ldlr^{-/-}CsfiCre^{+/-}GSK3\beta^{fl/fl}$	**LiβKO**	+	macrophage GSK3β$^{-/-}$	
$Ldlr^{-/-}CsfiCre^{+/-}GSK3\alpha^{fl/fl}GSK3\beta^{fl/fl}$	**LiαβKO**	+	macrophage GSK3α$^{-/-}$β$^{-/-}$	

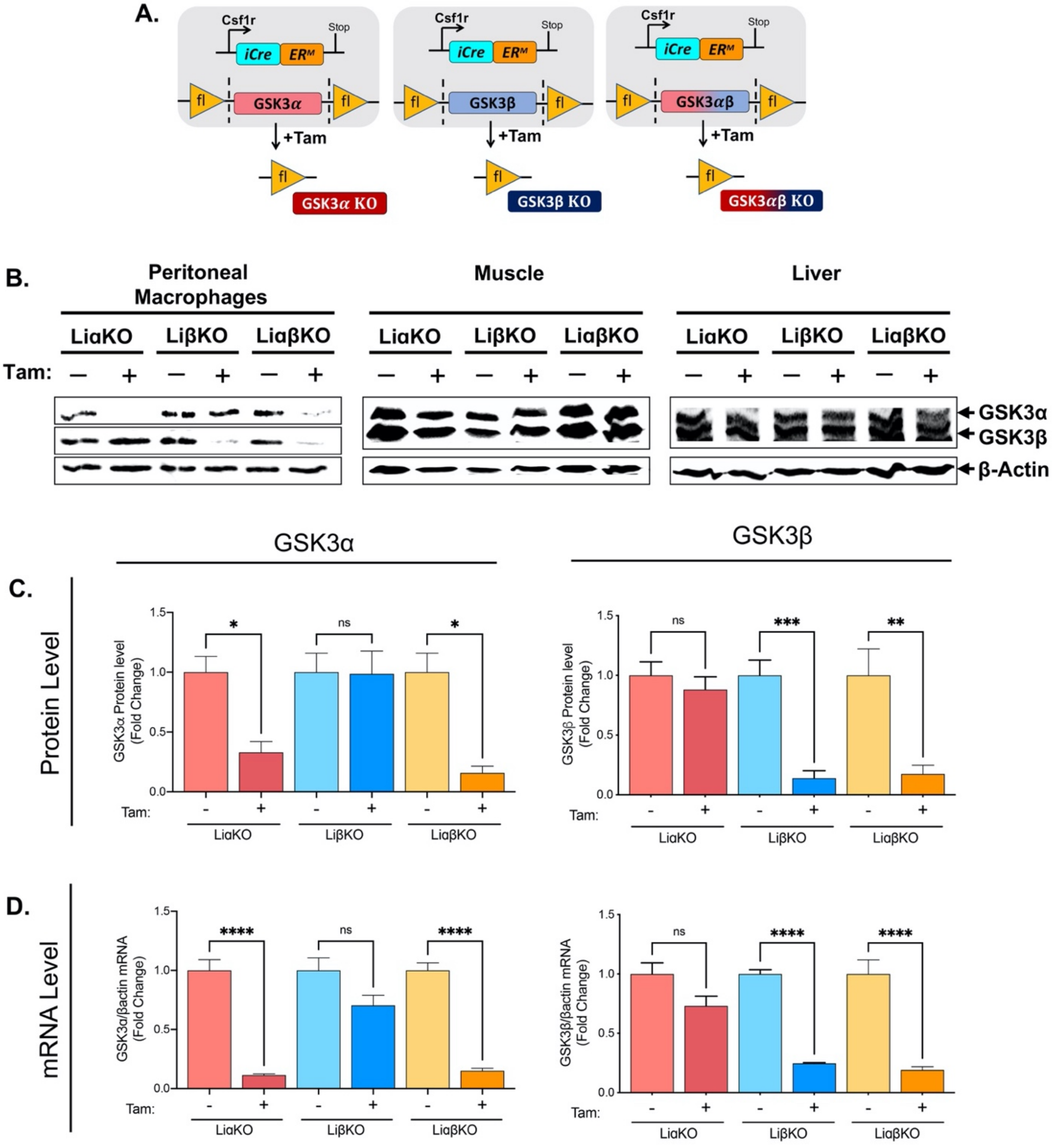

Supplementary Figure S1. Characterization of myeloid cell–specific GSK3α and/or GSK3β inducible knockout in macrophages derived from iCre Mice. **A.** Schematic diagram of transgenic mice crossing and tamoxifen induction of Cre activity. **B.** Whole tissue lysates from macrophages, muscle and liver from GSK3α and/or GSK3β iCre knockout mice, and controls were resolved by SDS-PAGE and probed for GSK3α and GSK3β protein. Quantified GSK3α and GSK3β **C.** protein levels and **D.** mRNA levels in macrophages determined by densitometry analysis. Results are reported as the fold change relative to non tamoxifen treatment. n=4; mean ± SEM; *p<0.05, **p<0.01, ***$p < 0.001$, ****$p < 0.0001$. Tam, tamoxifen.

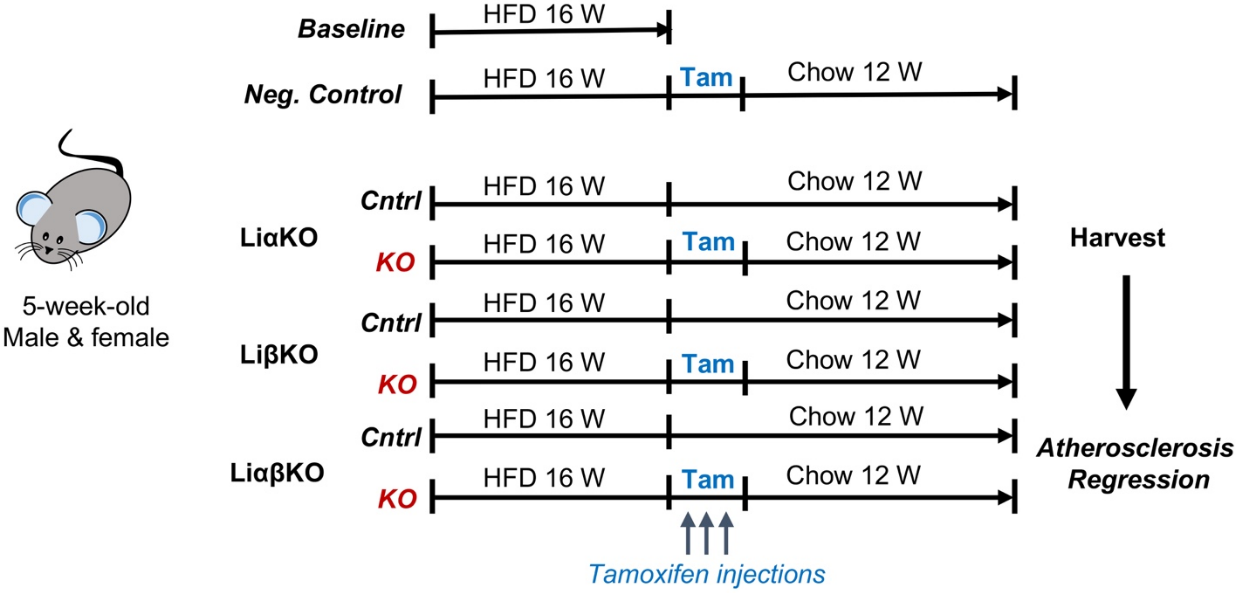

Experimental Mice: (n=9-13 each group)

Baseline (*$Ldlr^{-/-}$ $Csf1riCre^{+/-}$ $GSK3\alpha^{fl/fl}/\beta^{fl/fl}/\alpha^{fl/fl}\beta^{fl/fl}$***)**
Neg. Control (*$Ldlr^{-/-}$ $Csf1riCre^{-/-}$ $GSK3\alpha^{fl/fl}/\beta^{fl/fl}/\alpha^{fl/fl}\beta^{fl/fl}$***)**
LiαKO *($Ldlr^{-/-}$$Csf1riCre^{+/-}$$GSK3\alpha^{fl/fl}$)*
LiβKO *($Ldlr^{-/-}$ $Csf1riCre^{+/-}$ $GSK3\beta^{fl/fl}$)*
LiαβKO *($Ldlr^{-/-}$ $Csf1riCre^{+/-}$ $GSK3\alpha^{fl/fl}\beta^{fl/fl}$)*

Supplementary Figure S2. Experimental Design to study the effects of Tamoxifen triggered GSK3α and/or GSK3β deficiency on atherosclerotic plaque regression.

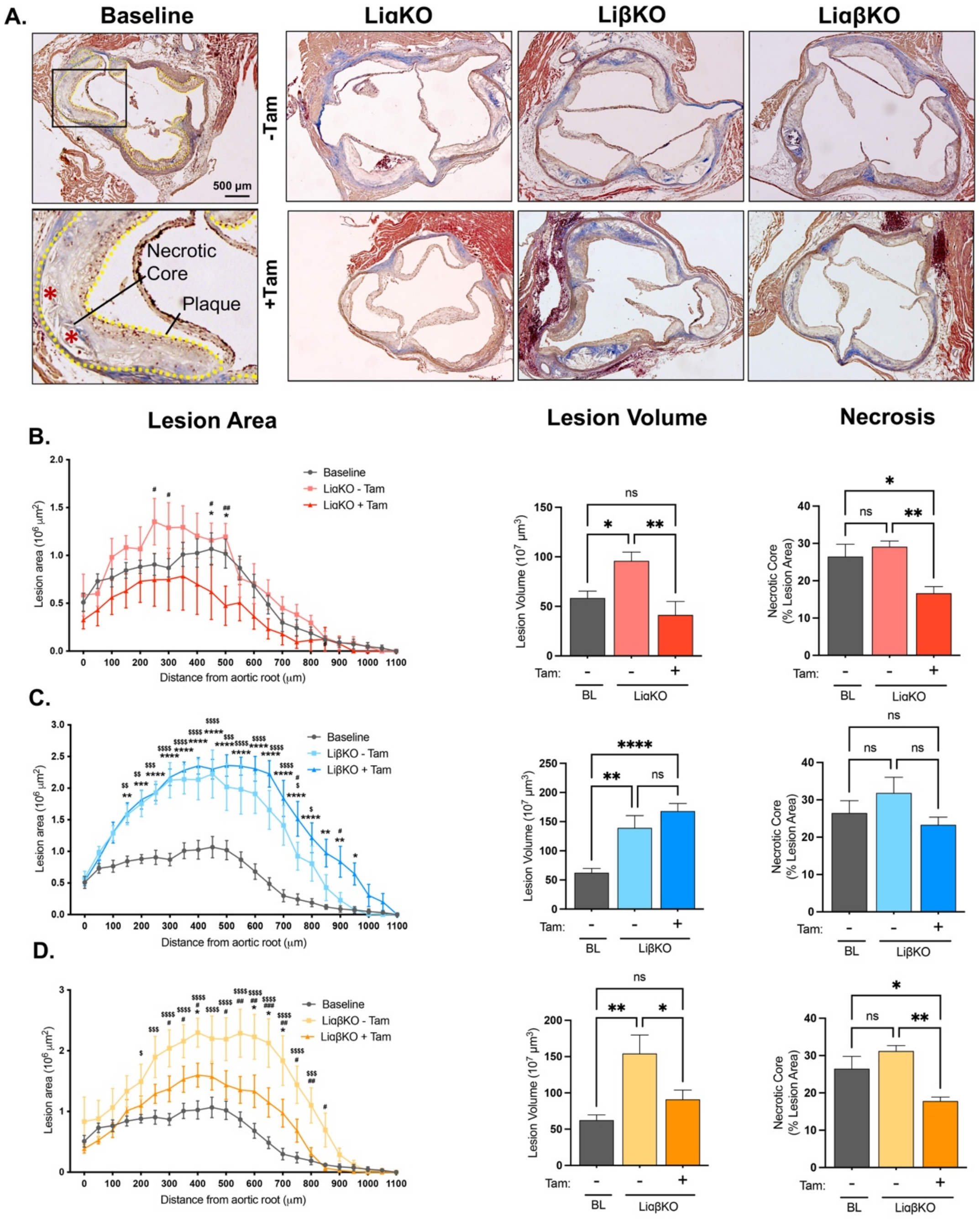

Supplementary Figure S3. Atherosclerosis regression in Male tamoxifen induced GSK3α and/or GSK3β deficient male Ldlr$^{-/-}$ mice. **A.** Representative aortic root sections from high-fat diet (HFD) fed baseline, LiαKO, LiβKO, LiαβKO mice with and without tamoxifen (Tam) treated mice stained with Masons trichrome. Quantification of atherosclerotic lesion area, lesion volume and % necrotic area at the aortic sinus and ascending aorta in **B.** LiαKO, **C.** LiβKO, and **D.** LiαβKO mice. n=5-8; mean ± SEM; **C.*** is the comparison to baseline and **#** is the comparison between WT and KO ; *p<0.05, **p<0.01, ***p < 0.001,**** p < 0.0001. Tam, tamoxifen; BL, baseline.

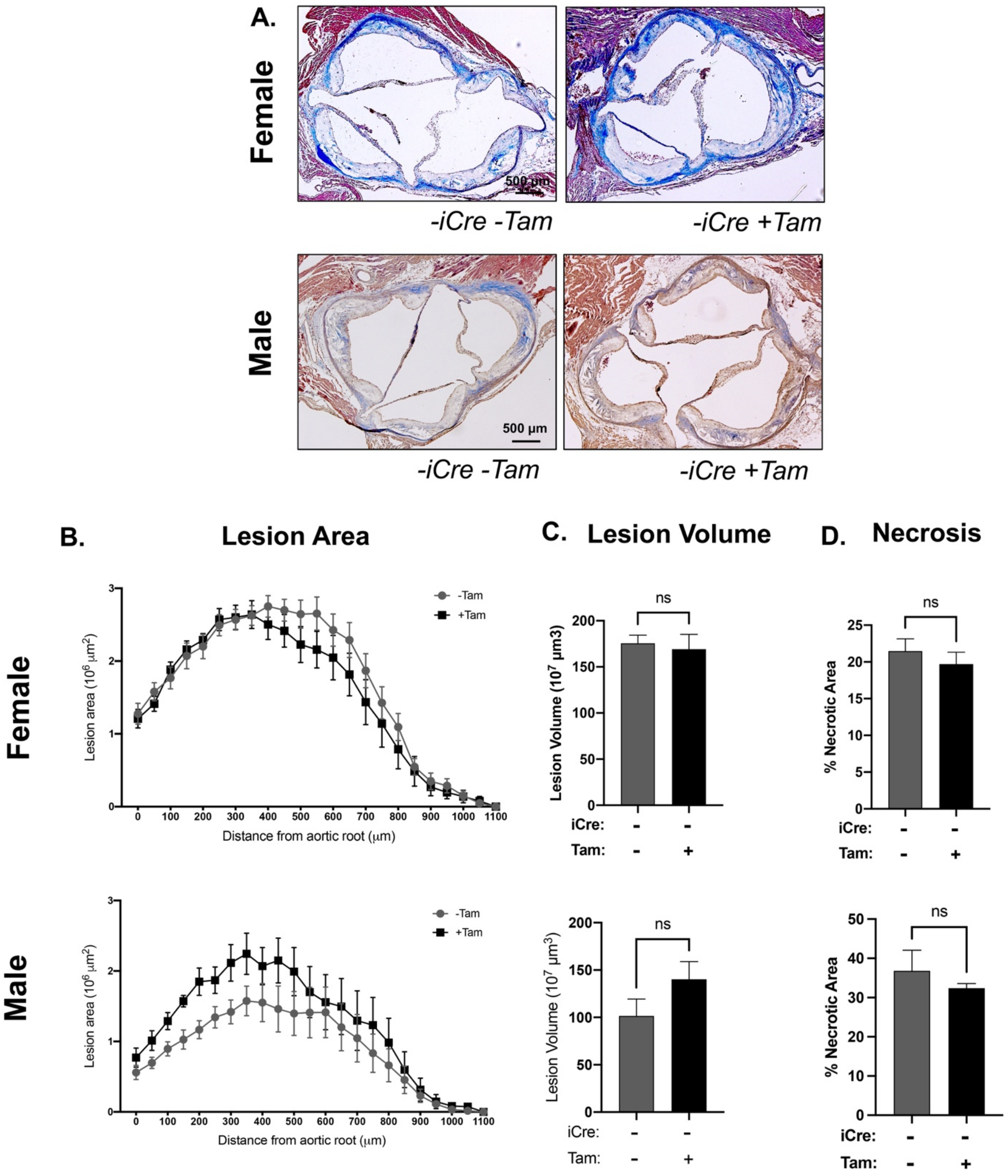

Supplementary Figure S4. Atherosclerosis regression in tamoxifen induced Ldlr$^{-/-}$ mice. In female **A.** Representative aortic root sections from high-fat diet (HFD) fed baseline and tamoxifen (Tam) treated negative control mice stained with Masons trichrome. Quantification of atherosclerotic **B.** lesion area and **C.** lesion volume at the aortic sinus and ascending aorta. **D.** Quantification of % necrotic area. n=9-13; mean ± SEM. Tam, tamoxifen.

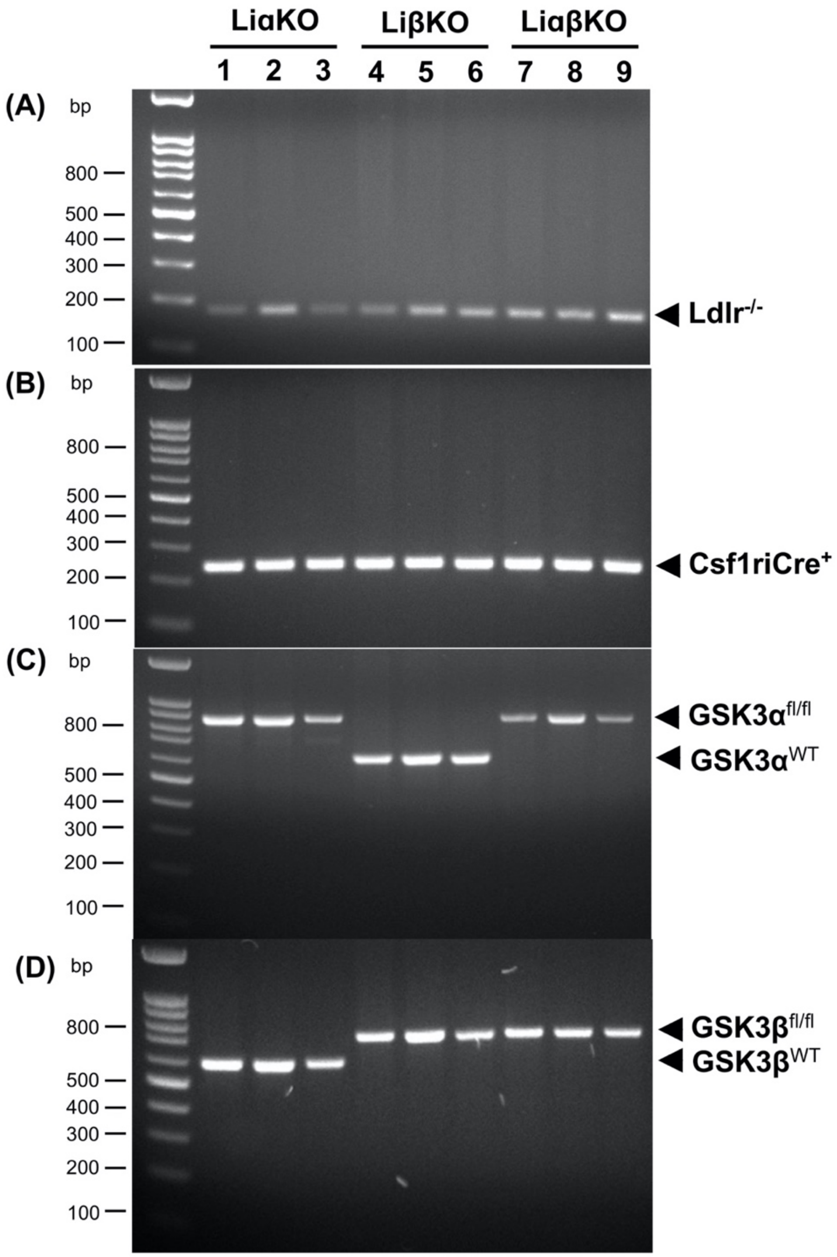

Supplementary Figure S5. PCR analysis of mouse models(Genotype). Genotyping of LiαKO, LiβKO, and LiαβKO mice. **A.** indicates $Ldlr^{-/-}$ (~179bp band) **B.** indicates $Csf1riCre^{+}$ (~200bp band). **C.** indicates $GSK3\alpha^{fl/fl}$ (~750bp band) or $GSK3\alpha^{WT}$ (~600bp band). **D.** indicates $GSK3\beta^{fl/fl}$ (~685bp band) or $GSK3\beta^{WT}$ (~585bp band)

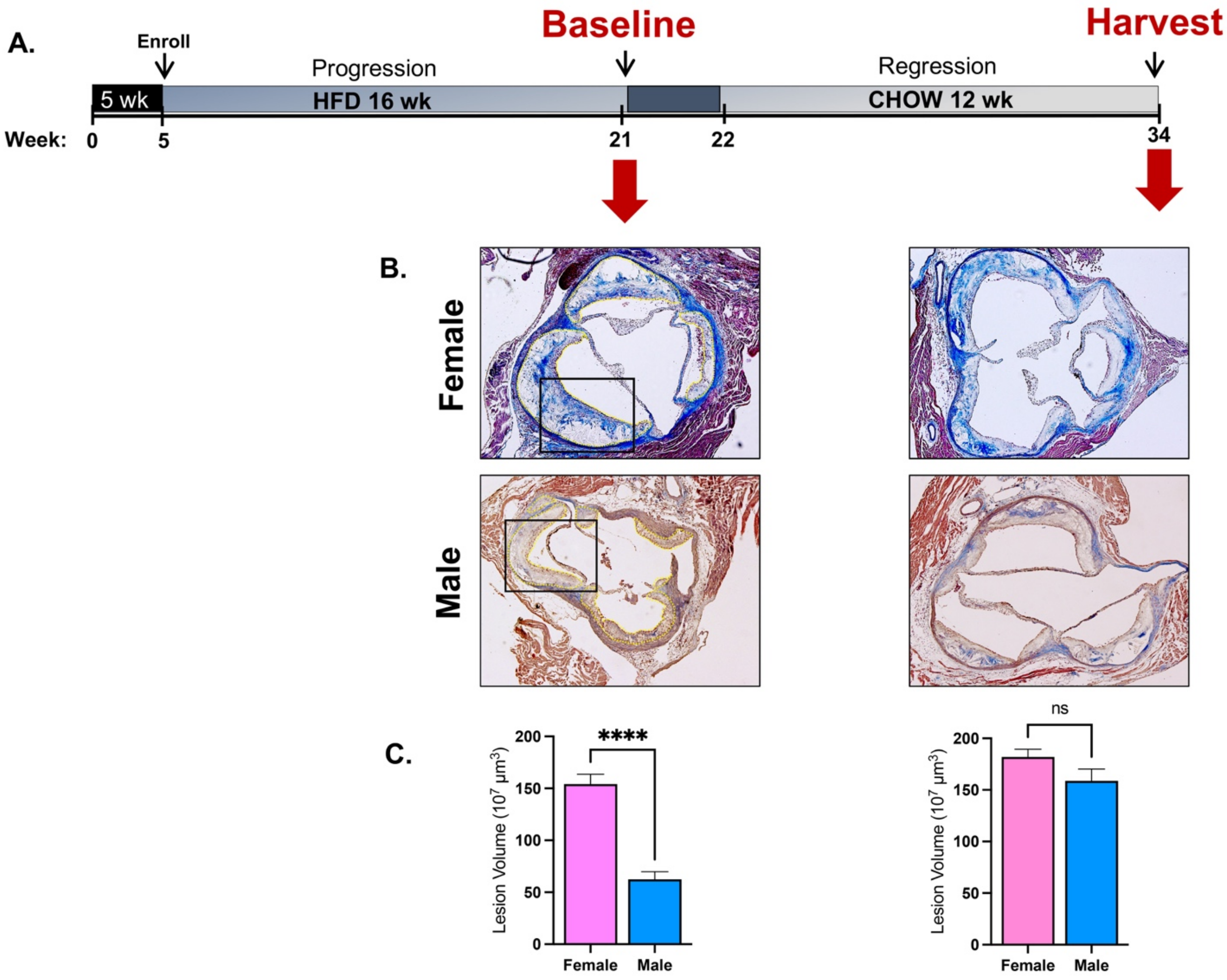

Supplementary Figure S6: Male and female *Ldlr*$^{-/-}$ mice express atherosclerosis differently at aortic root. A. Experimental design. **B.** Representative aortic root sections from female and male *Ldlr*$^{-/-}$ mice after 16 weeks of high fat diet (Baseline) and at a time harvest (end of study). **C.** Quantification of atherosclerotic lesion lesion volume at the aortic sinus and ascending aorta.

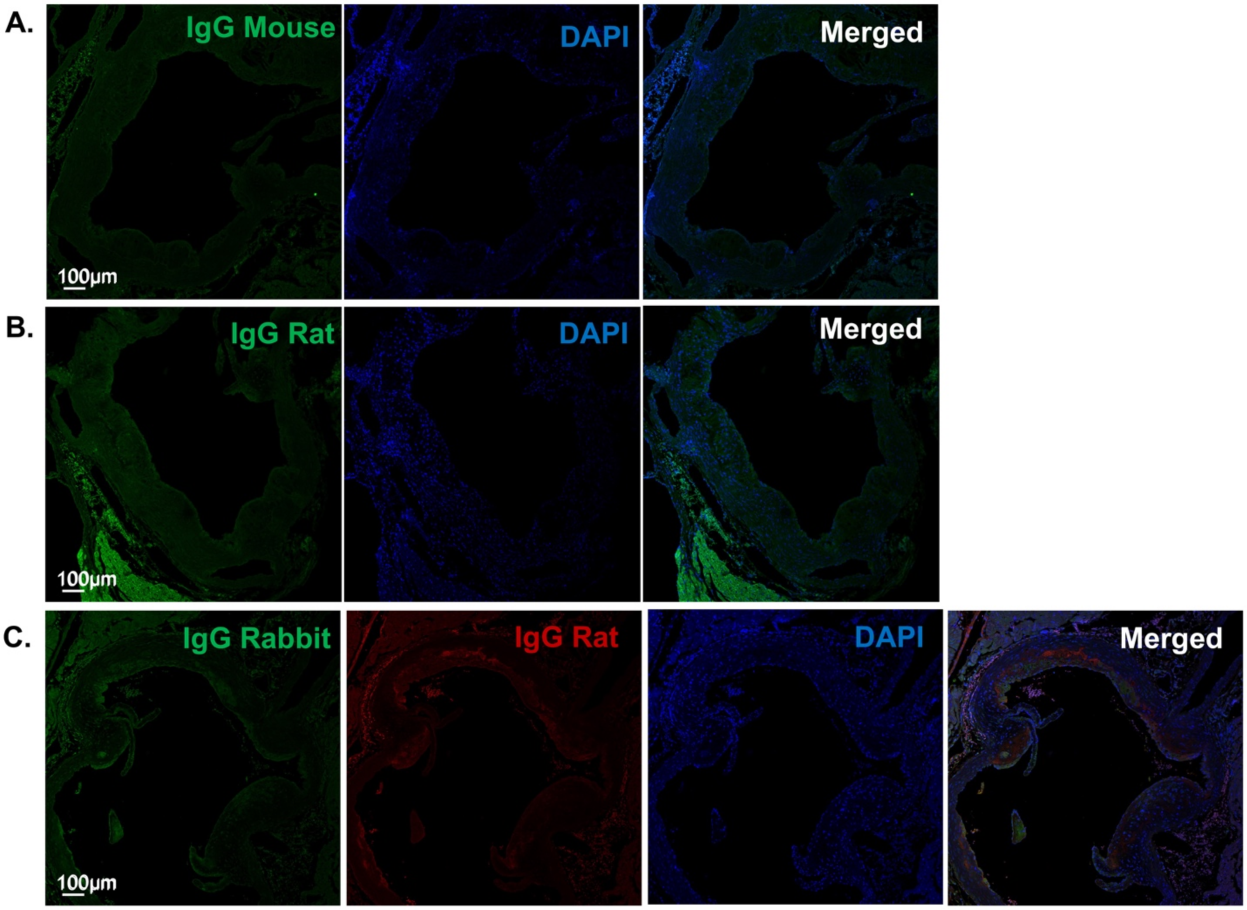

Supplementary Figure S7. Negative Controls for immunofluorescent staining. Representative aortic root sections stained with with pre-immune IgG antibodies to control for non-specific binding. Representative images of control IgG immunofluorescent staining and specific secondary antibody staining for **A.** anti-mouse, **B.** anti-rat, **C**. co-stained anti-rabbit and anti-rat. DAPI nuclear counterstaining is shown in blue.
